# Women’s knowledge of and attitudes toward cervical cancer and cervical cancer screening in Zanzibar, Tanzania: a cross-sectional study

**DOI:** 10.1186/s12885-020-6528-x

**Published:** 2020-01-28

**Authors:** Qiao Weng, Jie Jiang, Fatma Mrisho Haji, Lamlet Hassan Nondo, Huaijun Zhou

**Affiliations:** 1 Department of Obstetrics & Gynecology, Nanjing Drum Tower Hospital, Affiliated to Nanjing University Medical College, Nanjing, 210008 China; 2 Department of Obstetrics & Gynecology, Drum Tower Clinical Medical College, Nanjing Medical University, Nanjing, 210008 China; 3 Jiangsu Province Center for Disease Prevention and Control, Nanjing, China; 4 Department of Obstetrics & Gynecology, Mnazi Mmoja Hospital, Zanzibar, Tanzania

**Keywords:** Knowledge, Cervical cancer, Screening, Zanzibar

## Abstract

**Background:**

Cervical cancer is the most common cancer and the leading cause of cancer death among women in Tanzania. Knowledge of and willingness to receive a cervical cancer screening are important determinants of prevention. This study aimed to describe women’s awareness of cervical cancer and to explore the attitudes toward, acceptability of and barriers to cervical cancer screening (CCS) in Zanzibar.

**Methods:**

A cross-sectional study was conducted from March to June 2018 involving 1483 women from 10 districts in Zanzibar who responded to questionnaires concerning their general demo-graphic characteristics, screening willingness and awareness of cervical cancer. Chi-square tests, analysis of variance (ANOVA) and stepwise multiple regression were conducted using STATA 15.1 software.

**Results:**

The average total knowledge score (TKS) was 7.84 ± 5.32 on a 23-point scale. Educational level and family income were positively correlated with the TKS. Previous schistosomiasis history and family genetic disease history were strong predictors of screening willingness. Women were less likely to be screened freely if they had 7 or more deliveries and were unaware of any previous family tumor history. Age and educational level were negatively associated non-free screening willingness, while family income was positively associated; being divorced/widowed or single and being unaware of any previous family tumor history were predictors of screening reluctance, while previous disease history was a strong predictor of non-free screening willingness. Fear of screening and inconvenience were the primary concerns among the Zanzibari interviewees. Compared to the 20–49 age group, more women in the less than 20 and 50 or more age groups thought cervical cancer screening was not necessary. The highest rate of cognitive accuracy in regard to cervical cancer warning signs and risk factors was only 37.76%.

**Conclusions:**

The findings revealed that knowledge of cervical cancer was poor. Educational level, family income and awareness of previous disease history were significant influencing factors of screening uptake. Specific awareness programs to increase knowledge of cervical cancer and screening willingness should be designed and implemented in the public without delay, especially for younger and elderly women.

## Background

Cervical cancer, with an estimated 570,000 new cases and 311,000 deaths in 2018 globally, is the fourth most common cancer and the fourth leading cause of cancer death among women world-wide; however, it is the most commonly diagnosed cancer in 28 countries and the leading cause of cancer-related death in 42 countries, the majority of which are in Sub-Saharan Africa [1]. Tanzania exhibits the sixth highest rate of cervical cancer in the world, with an age-standardized incidence of 59.1 per 100,000 women and an age-standardized mortality of 42.7 per 100,000 women per year. Cervical cancer was responsible for 39% of all newly developed cancers in women, and among them, approximately 80% presented with advanced-stage cancer; thus, cervical cancer was the leading cause of cancer related death among Tanzanian women [1]. The lack of available comprehensive screening programs for cervical cancer helps explain the shockingly high incidence and mortality in Sub-Saharan African countries, and it has already been shown that an organized screening program can reduce incidence and mortality by 80% [2].

In Tanzania, due to inadequate personnel and deficiencies in the health system infrastructure, cervical cancer prevention remains largely opportunistic, often relying on low-resource visual inspection with acetic acid (VIA) [3]. However, the reported uptake of this screening service remains low, suggesting that there are barriers preventing women from being screened [4]. A general lack of awareness of and insufficient preparations for screening programs both contribute to ineffective screening results. A recent study from the Kilimanjaro region of Tanzania reported that only 6% of women had ever been screened for cervical cancer, while the majority of women perceived that they were susceptible to cervical cancer and were willing to accept screening if it were made available [5]. Thus, it is necessary to understand and address the multifaceted health beliefs that are likely to influence women’s willingness to schedule and obtain screening.

In Zanzibar, a semiautonomous region of Tanzania, information about the incidence and mortality of cervical cancer and about women’s knowledge about cervical cancer has never been report-ed. Assessing women’s knowledge of cervical cancer will help in taking into account the actual scenario and in identifying approaches to increase the perception of cervical cancer or to change attitudes toward cervical cancer screening and create demand for cervical cancer screening services. The purpose of this study, therefore, was to describe women’s awareness of cervical cancer and to explore the attitudes toward, acceptability of and barriers to cervical cancer screening (CCS) in a population-based sample of women living in Zanzibar to better organize the forthcoming China-Zanzibar cervical cancer screening program in Zanzibar.

## Methods

### Study design and respondents

A cross-sectional survey was conducted from March 2018 to June 2018 among women aged 14–65 years old in Zanzibar. Based on data from the Zanzibar Population Registry, a 95% confidence interval with a margin error of 3% was employed in the following formula [6] to obtain a conservative sample size of 1066:

$$ \mathsf{n}={\mathsf{t}\mathsf{\alpha}}^{\mathsf{2}}\ast \mathsf{p}\ast \mathsf{q}\ast \mathsf{N}/\left[\left(\mathsf{N}-\mathsf{1}\right)\ast {\mathsf{e}}^{\mathsf{2}}+{\mathsf{t}\mathsf{\alpha}}^{\mathsf{2}}\ast \mathsf{p}\ast \mathsf{q}\right] $$

Here, n is the sample size; tα is the value of the normal curve associated with the confidence level; p is the expected percentage of the response variable; q equals 1-p; e is the margin of error; and N is the population size.

With a total of 10 administrative districts involved, we randomly chose 5 wards from each district and randomly visited 30 women from each ward. Assisted by local volunteers from the 10 districts, we interviewed 1500 women at various community sites in the wards, including mosques, grocery stores, health fairs, community centers and homes. As there were 17 non-respondents, 1483 participants were involved in our study.

### Procedures

The study questionnaire was designed in English and translated, and an interviewer administered it in Swahili, the official language of Zanzibar. All employees received a three-page questionnaire, with the first page explaining the purpose and importance of the study. Trained volunteers gave a brief introduction with regard to the purpose of the study before both voluntary oral and written consent were obtained. The participants were assured of complete anonymity in regard to the answers they provided. Most items were closed-response questions, and the questionnaire took approximately 30 min to complete. There were no pre-cervical cancer screening services in the implementing areas during this study, which was a baseline assessment before the introduction of screening services. Brief education on the location and function of the cervix was provided during the survey for women who said they had never heard the word before.

### Measures

The survey (Additional file 1) was developed by integrating validated questions from both the Cervical-Cancer-Knowledge-Prevention-64 (CCKP-64) [7] and the Cervical Cancer Awareness Measure (CAM) questionnaires [8]. The final questionnaire consisted of 33 items of general demographic questions, 3 closed-ended questions that determined attitudes toward screening and 14 closed-ended questions that determined awareness of cervical cancer. Questions were chosen based on their relevance to the cultural setting, considering the diversity of cultural and religious beliefs in Zanzibar. The questionnaire was structured into two sections. The background section mainly included demographic data, lifestyle-related factors such as drinking and eating habits, previous history of dis-eases, experience of gynecological examination and women’s attitudes toward screening. The other section assessed knowledge of cervical cancer and cervical cancer screening using a multiple-choice format. The questions were adopted from previous studies [9] with questions on awareness and understanding of cervical cancer and CCS, including signs and symptoms, risk factors and women’s attitudes toward the disease. The structured questionnaire was used by five trained interviewers. To increase the reliability of the information, the interviewers were trained to administer the questionnaires in a uniform way to prevent their own interpretation of the questions.

To assess the participants’ overall level of awareness of cervical cancer and CCS, total knowledge scores (TKSs) were calculated based on the answers to the questions of the second section. For each correct answer or each positive response, a score of one point was obtained, and the sum of the points in the questionnaire was a total score of 23 points. A higher score corresponds to greater knowledge of cervical cancer and screening.

### Data analysis

The data were analyzed using STATA software version 15.1. Basic descriptive statistics and frequency calculations were performed for all variables. In bivariate models for identifying the factors associated with screening and cervical cancer awareness, Pearson chi-square or Fisher’s exact tests and one-way analysis of variance (ANOVA) with Bonferroni pairwise adjustment were used to compare the frequencies and continuous variables among groups. To gain insights into the independent effects of characteristics on screening willingness and knowledge scores, multiple logistic and linear regression models using a backward stepwise procedure were applied as supplementary analyses. A two-sided *P* < 0.05 was considered statistically significant, and *P* values with significance are marked in bold in the tables.

### Ethical considerations

Ethical clearance for this study was obtained from the Zanzibar Medical Research and Ethics Committee (reference number ZAMREC/0003/APRIL/ 2018). Informed consent forms were signed by the interviewer, indicating that the study objectives were explained to the participants and that both verbal and written consent were received. Confidentiality was ensured throughout the process of data collection. The analyses were performed using identified code numbers rather than participant names.

## Results

### Sociodemographic characteristics

The characteristics of the participants are shown in Table 1. Of the 1483 women, the mean age was 32.86 years (SD 10.93, range 14 to 65), and 85.5% of the participants were aged 20 to 49 years. Nearly all of the interviewees were in the Muslim region (98.04%). The vast majority of the participants were married or cohabiting (74.58%), whereas 14.95% were identified as consanguineous mating. Nearly one quarter (23.40%) of the women had had sexual onset before 18 years of age, and the average parity of all respondents was 2.96, with a maximum of 14. Over half (62.04%) had obtained a secondary level of education, and approximately two-thirds (66.01%) lived lives of bare subsistence. Most women (80.38%) drank tap water; 16.5% drank well water, and 3.44% drank pure water. A considerable number of respondents drank local spice tea frequently (39.04%) or occasionally (27.78%). Approximately one-third (34.66%) of the participants had a previous disease history, including schistosomiasis history, which accounted for 7.28% of all women in the sur-vey. Genetic diseases affected 15.31% of women in our study, and 9.78% of participants had a family cancer history. A total of 1304 women (87.93%) were willing to attend a free screening pro-gram, while only 852 (57.45%) were willing to uptake a screening at their own expense. Only 4.38% of the respondents had previously received cervical cancer screening. The average TKS was 7.84 ± 5.32 (range from 0 to 22).

**Table 1:** Socio-demographic characteristics of participants

Variable	N (%)	Mean ± SD
Age group (y)		32.86 ± 10.93
< 20	82 (5.53)	
20–29	602 (40.59)	
30–49	666 (44.91)	
≥ 50	130 (8.77)	
Missing	3 (0.2)	
Ethnicity		
Muslim	1454 (98.04)	
Jesus	25 (1.69)	
Other	2 (0.13)	
Missing	2 (0.13)	
Marital States		
Married	1070 (72.15)	
^a^Consanguineous married	160 (14.95)	
Cohabiting	36 (2.43)	
Divorced/Widowed	145 (9.78)	
Single	232 (15.64)	
Parity		2.97 ± 2.81
0	359 (24.21)	
1–3	583 (39.31)	
4–6	363 (24.48)	
≥ 7	176 (11.87)	
Missing	2 (0.13)	
Education level		
No formal	125 (8.43)	
Primary	262 (17.67)	
Secondary	920 (62.04)	
Tertiary	176 (11.87)	
First Sex Age(y)		19.72 ± 4.30
< 15	94 (6.34)	
15–17	253 (17.06)	
18–24	719 (48.48)	
≥ 25	157 (10.59)	
None	260 (17.53)	
Family Income		
Wealthy	8 (0.54)	
Just getting by	979 (66.01)	
Poor	496 (33.45)	
Drinking Water		
Well	238 (16.05)	
Tap	1192 (80.38)	
Pure	51 (3.44)	
Missing	2 (0.13)	
Spice tea		
Often	579 (39.04)	
Occasionally	412 (27.78)	
Rare	403 (27.17)	
Never	89 (6)	
Disease History		
None	942 (63.52)	
Schistosomiasis	108 (7.28)	
Other Disease	406 (27.38)	
Don’t know	27 (1.82)	
Genetic Disease		
None	1216 (82.00)	
Yes	227 (15.31)	
Don’t know	40 (2.7)	
Family Cancer History		
None	1231 (83.01)	
Yes	145 (9.78)	
Don’t know	107 (7.22)	
Previous Screening		
Yes	65 (4.38)	
No	1417 (95.55)	
Don’t know	1 (0.07)	
Willingness for free screening		
Yes	1304 (87.93)	
No	129 (8.7)	
Don’t know	50 (3.37)	
Willingness for non-free screening		
Yes	852 (57.45)	
No	384 (25.89)	
Don’t know	247 (16.66)	
Knowledge Scores		7.84 ± 5.32

^a^Refers to the number of consanguineous marriage and the corresponding ratio to the married women

### Bivariate models for identifying the factors associated with screening and cervical cancer awareness

As presented in Table 2, women’s screening willingness was significantly associated with age group, marital status, parity, educational level, family income, personal history of disease, genetic disease and family cancer history. Participants between 20 and 49 years old showed higher acceptance of free screening (*P* = 0.001) and non-free screening (*P* = 0.042). The married/cohabiting group showed higher acceptance than the divorced/widowed group (60.58% vs 48.97%, *P* = 0.000). Women who had delivered 1–3 children were more likely to uptake both types of screening than the highest parity group (90.74% vs 78.98%, *P* = 0.000 and 60.55% vs 53.98%, *P* = 0.000, respectively). There was no difference in the acceptance of free screening, but women without formal education were more likely to uptake non-free screening than those who obtained a tertiary level of education (64.8% vs 50%, *P* = 0.000). Wealthy women were more willing to uptake non-free screening than needy women (62.5% vs 50.81%, *P* = 0.000). Women with previous disease, especially those with a history of schistosomiasis, were more likely to accept both types of screening than healthy women (99.07% vs 85.99%, *P* = 0.001; 93.52% vs 51.38%, *P* = 0.000). Women who had genetic disease were more willing to uptake free screening (95.15% vs 86.35%, P = 0.000); however, for self-paying screening, there was no difference between the two groups. Moreover, women who were unaware of any family tumor history were less likely to uptake any type of screening than other women (*P* = 0.042 & *P* = 0.005).

**Table 2:** Bivariate models for identification of factors associated with screening and cervical cancer awareness

Variable		Free Screening		Non-free Screening		Knowledge Scores	
	N	N(%)	*P*	N(%)	*P*	N(%)	*P*
Age (y)							
< 20	82	65 (79.27)	**0.001**	42 (51.22)	**0.042**	9.13 ± 5.86	0.071
20–29	602	540 (89.70)		363 (60.30)		7.97 ± 5.55	
30–49	666	591 (88.74)		378 (56.46)		7.65 ± 4.93	
≥ 50	130	105 (80.77)		68 (52.31)		7.38 ± 5.74	
Ethnicity							
Muslim	1454	1276 (87.76)	0.607	831 (57.15)	0.195	7.87 ± 5.34	0.133
Jesus	25	24 (96)		20 (80)		5.72 ± 3.39	
Other	2	2 (100)		1 (50)		8.5 ± 7.78	
Marital States							
Married/Cohabiting	1106	981 (88.70)	0.061	670 (60.58)	**0.000**	7.64 ± 5.21	**0.000**
Divorced/Widowed	145	121 (83.45)		71 (48.97)		6.96 ± 5.10	
Single	232	202 (87.07)		111 (47.84)		9.31 ± 5.70	
Parity							
0	359	313 (87.19)	**0.000**	191 (53.20)	**0.000**	8.57 ± 5.43	**0.014**
1–3	583	529 (90.74)		353 (60.55)		7.72 ± 5.18	
4–6	363	321 (88.43)		211 (58.13)		7.69 ± 5.41	
≥ 7	176	139 (78.98)		95 (53.98)		7.12 ± 5.22	
Education level							
No formal	125	107 (85.60)	0.216	81 (64.80)	**0.000**	5.36 ± 5.57	**0.000**
Primary	262	227 (86.64)		162 (61.83)		6.93 ± 5.54	
Secondary	920	810 (88.04)		521 (56.63)		7.97 ± 5.03	
Tertiary	176	160 (90.91)		88 (50)		10.27 ± 5.24	
First Sex Age (y)							
< 15	94	76 (80.85)	0.190	56 (59.57)	0.124	6.88 ± 5.13	**0.012**
15–17	253	222 (87.75)		155 (61.26)		6.83 ± 5.42	
18–24	719	634 (88.18)		414 (57.58)		7.90 ± 5.13	
≥ 25	157	144 (91.72)		95 (60.51)		8.11 ± 5.54	
Family Income							
Wealthy	8	6 (75)	0.270	5 (62.50)	**0.000**	10 ± 4.44	**0.000**
Just getting by	979	863 (88.15)		595 (60.78)		8.34 ± 5.12	
Poor	496	435 (87.70)		252 (50.81)		6.81 ± 5.57	
Disease History							
None	942	810 (85.99)	**0.001**	484 (51.38)	**0.000**	8.11 ± 5.14	**0.000**
Schistosomiasis	108	107 (99.07)		101 (93.52)		3.21 ± 6.30	
Other Disease	406	362 (89.16)		246 (60.59)		8.62 ± 4.93	
Genetic Disease							
None	1216	1050 (86.35)	**0.000**	683 (56.17)	0.108	7.83 ± 5.33	0.256
Yes	227	216 (95.15)		138 (60.79)		8.27 ± 5.47	
Family Cancer History							
None	1231	1090 (88.55)	**0.042**	717 (58.25)	**0.005**	7.64 ± 5.33	**0.004**
Yes	145	128 (88.28)		91 (62.76)		9.05 ± 5.00	
Don’t know	107	86 (80.37)		44 (41.12)		8.51 ± 5.47	
Previous Screening							
Yes	65	60 (92.31)	0.490	815 (57.52)	0.720	10.28 ± 4.75	**0.000**
No	1417	1244 (87.79)		37 (56.92)		7.72 ± 5.32	

The knowledge scores were correlated with marital status, parity, educational level, age of sexual onset, family income, personal previous disease, family cancer history and previous screening. Women who were unmarried, were nulliparous, had obtained tertiary education, had sexual onset after 24 years of age and were wealthy scored higher compared to their counterparts (*P* < 0.05). Women with previous schistosomiasis history were much less aware than those with other disease histories (3.21 ± 6.30 vs 8.62 ± 4.93, *P* = 0.000). Higher awareness also appeared to be associated with family cancer history (9.05 ± 5.00 vs 7.64 ± 5.33, *P* = 0.004) and previous screening (10.28 ± 4.75 vs 7.72 ± 5.32, *P* = 0.000).

### Multivariate models for identifying the factors associated with screening and cervical cancer awareness

In the multivariate logistic regression model, previous schistosomiasis history (OR = 24.14, 95% CI = 3.31–176.27) and family genetic disease history (OR = 3.14, 95% CI = 1.64–6.00) were strong predictors of free screening willingness. Women were less likely to uptake free screening if they had 7 or more deliveries (OR = 0.30, 95% CI = 0.15–0.60) and were unaware of any previous family tumor history (OR = 0.38, 95% CI = 0.22–0.67) (Table 3).

**Table 3:** Multivariate models for identification of factors associated with willingness of free screening

Willingness to uptake Free Screening	OR	95%CI	*P*
Age	1.11	0.91–1.37	0.310
Parity (Ref: 0)			
1–3	1.23	0.78–1.94	0.369
4–6	0.80	0.44–1.45	0.465
≥ 7	0.30	0.15–0.60	**0.001**
Previous disease (Ref: None)			
Other	1.26	0.86–1.85	0.235
Unknown	2.01	0.38–10.68	0.412
Schistosomiasis	24.14	3.31–176.27	**0.002**
Family tumor history (Ref: None)			
Yes	0.91	0.52–1.59	0.734
Unknown	0.38	0.22–0.67	**0.001**
Family genetic disease (Ref: None)			
Yes	3.14	1.64–6.00	**0.001**
Unknown	3.93	0.78–19.90	0.098

*Abbreviations*: *OR* Odds Ratio, *CI* Confidence interval, *Ref* Reference

As shown in Table 4, age and educational level were negatively associated and family income was positively associated with willingness to pay for screening; being divorced/widowed or single and being unaware of any previous family tumor history were predictors of not up-taking self-paying screening, while previous disease history was a strong predictor of non-free screening willingness.

**Table 4:** Multivariate models for identification of factors associated with willingness of non-free screening

Willingness to uptake Non-free Screening	OR	95%CI	*P*
Age	0.84	0.73–0.96	**0.014**
Marriage (Ref: Married/Cohabiting)			
Divorced/Widowed	0.64	0.43–0.94	**0.025**
Single	0.47	0.30–0.73	**0.001**
Parity (Ref: 0)			
1–3	0.98	0.67–1.43	0.922
4–6	0.87	0.56–1.35	0.542
≥ 7	0.60	0.35–1.02	0.06
Education level	0.80	0.68–0.95	**0.009**
Family income	1.79	1.41–2.26	**< 0.001**
Family tumor history (Ref: None)			
Yes	1.30	0.90–1.89	0.167
Unknown	0.39	0.24–0.63	**< 0.001**
Previous disease (Ref: None)			
Other	1.58	1.23–2.03	**< 0.001**
Unknown	7.19	2.61–19.84	**< 0.001**
Schistosomiasis	17.89	8.04–39.84	**< 0.001**

*Abbreviations*: *OR* Odds Ratio, *CI* Confidence interval, *Ref* Reference

Table 5 present the factors that may be linearly dependent on the TKS using multiple linear regressions. Among these variables, educational level and family income were significant TKS predictors (Coef = 1.08, 95% CI = 0.69–1.46 and Coef = 0.81, 95% CI = 0.26–1.36, respectively). Single women scored more compared to married and cohabited women (Coef = 2.15, 95% CI = 0.72–3.58). Women who had 7 or more deliveries were more likely to have a higher score (Coef = 1.37, 95% CI = 0.21–2.53) compared to those who had never delivered. In addition, women with family tumor history and cervical screening history were associated with higher knowledge scores (Coef = 1.07, 95% CI = 0.20–1.94 and Coef = 2.40, 95% CI = 1.14–3.66, respectively). Women with previous schistosomiasis history and who were unaware of any previous disease history scored lower compared to healthy participants (Coef = − 3.95, 95% CI = -4.99–2.90 and Coef = − 4.05, 95% CI = -6.13–1.97, respectively).

**Table 5:** Multivariate models for identification of factors associated with TKS

TKS	Coef.	95%CI	*P*
Marriage (Ref: Married/Cohabiting)			
Divorced/Widowed	0.01	−0.91-0.93	0.980
Single	2.15	0.72–3.58	**0.003**
Education	1.08	0.69–1.46	**< 0.001**
Parity (Ref: 0)			
1–3	0.51	−0.38-1.39	0.264
4–6	0.91	−0.07-1.88	0.068
≥ 7	1.37	0.21–2.53	**0.021**
Age of sexual onset (Ref: < 15)			
None	−0.10	−1.67-1.47	0.901
15–17	−0.02	−1.21-1.17	0.973
18–24	0.46	−0.65-1.57	0.416
≥ 25	0.47	−0.87-1.82	0.490
Family income	0.81	0.26–1.36	**0.004**
Family tumor history (Ref: None)			
Yes	1.07	0.20–1.94	**0.016**
Unknown	1.25	0.16–2.33	**0.024**
Previous disease (Ref: None)			
Other	0.34	−0.25-0.93	0.255
Unknown	−4.05	−6.13~ − 1.97	**< 0.001**
Schistosomiasis	−3.95	−4.99~ − 2.90	**< 0.001**
Previous screening (Ref: None)			
Yes	2.40	1.14–3.66	**< 0.001**

*Abbreviations*: *Coef* Coefficient, *CI* Confidence interval, *Ref* Reference

### TKS and distribution by different attitudes towards free and non-free screening

**Fig. 1:**
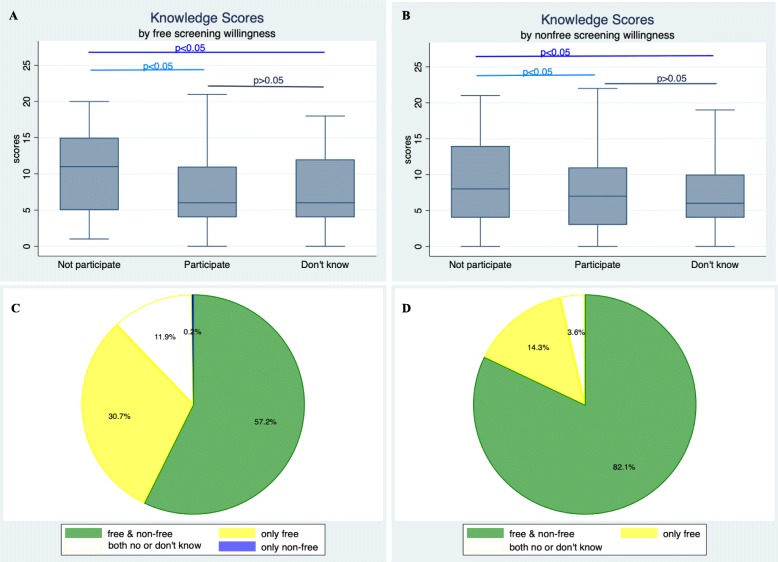
TKS and Distribution by different attitudes towards free and non-free screening. **a** Knowledge scores by free screening willingness; **b** Knowledge scores by non-free screening willingness; **c** All respondents’ attitude to uptake different screening; **d** Attitude of screening uptake in women scored 20 or more

As shown in Fig. 1, women who were not going to receive either free or non-free screening scored higher than the other two groups; however, there was a generally low level among all participants. Of all the women sampled, 87.9% were willing to receive free screening, and slightly more than half (57.4%) were willing to receive non-free screening; however, of the women who scored 20 points or more, nearly all (96.4%) were going to uptake free screening, and the majority (82.1%) were willing to uptake screening even at their own expense.

### Worries about cervical cancer screening among women of different age groups

Table 6 portrays the women’s worries about cervical cancer screening. Among the 1483 women who were interviewed, 548 instances of worry were expressed. Most of the worries were not significantly different between age groups; compared to the 20–49 age group, more women in the less than 20 and 50 or more age groups thought cervical cancer screening was not necessary.

**Table 6:** Worries for cervical cancer screening among women of different age groups

*Worries*	Number (*N* = 548)	Age group			*P*
		< 20	20–49	≥50	
*Fear to give a Pap-smear*	132	10 (12.20)	114 (8.99)	8 (6.15)	0.314
*Clinic is far away*	112	7 (8.54)	94 (7.41)	11 (8.46)	0.860
*Long appointment queues*	85	6 (7.32)	69 (5.44)	10 (7.69)	0.472
*It is not necessary*	49	5 (6.10)	35 (2.76)	9 (6.92)	**0.014**
*Fear the result*	30	0 (0.0)	28 (2.21)	2 (1.54)	0.357
*A recent health control at a gynecologist*	11	0 (0.0)	9 (0.71)	2 (1.54)	0.417
*Other reasons*	123	8 (9.76)	107 (8.44)	8 (6.15)	0.593

### Women’s awareness of cervical cancer warning signs and risk factors

**Fig. 2:**
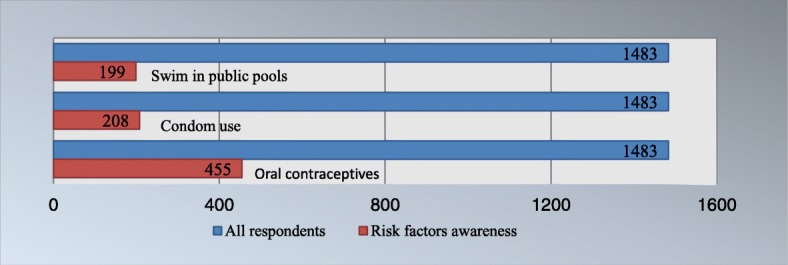
Women’s overworrying about cervical cancer risk factors

As shown in Table 7, the highest rate of cognitive accuracy is only 37.76%, with approximately four in ten denying that they knew anything about cervical cancer warning signs and risk factors (40.46 and 39.04%, respectively). Many women thought that oral contraceptives, condom usage and even swimming in public pools were risk factors for cervical cancer, with proportions of 30.68, 14.03 and 13.42%, respectively (Fig. 2).

**Table 7:** Frequency of correct answers for items about cervical cancer

*Questionnaire items*	*N (% answered correctly)* ^a^
Knowledge of Warning Signs for Cervical Cancer	
*Persistent vaginal discharge*	560 (37.76)
*Vaginal bleeding during or after sex*	484 (32.64)
*Vaginal bleeding between periods*	421 (28.39)
*Vaginal bleeding after menopause*	386 (26.03)
*Unexplained weight loss*	244 (16.45)
Knowledge of Risk Factors for Cervical Cancer	
*Many sexual partners*	539 (36.35)
*Sex at a young age*	482 (32.50)
*Sexually transmitted infection*	469 (31.63)
*Infection with HPV*	433 (29.20)
*Sexual partner with many other partners*	362 (24.41)
*Hypo immune function*	291 (19.62)
*Genetic factors*	204 (13.76)
*Rare health control*	191 (12.88)
*Not going for regular smear (Pap) tests*	139 (9.37)
*Miscarriages and abortions (Choose “No”)*	366 (24.68)

^a^Participants who answered “I don’t know” (600/1483 and 579/1483 respectively) were counted as incorrect responses

## Discussion

To the best of our knowledge, this cross-sectional survey research seems to be the first study employing a validated questionnaire to investigate the awareness of cervical cancer and the factors affecting attitudes toward screening among African women living in Zanzibar, Tanzania. The most important finding from this study indicates that women’s knowledge of cervical cancer was generally inadequate and was persistently associated with education, family income and family cancer history. Self-paying screening willingness was largely influenced by family income. Our findings are the first to uncover a strong association between schistosomiasis infection history and attitudes toward screening participation. Additionally, our study is the first to analyze women’s worries about cervical cancer screening among women of different age groups, reaching the conclusion that women in the younger age and older age groups in Zanzibar should be the focus of knowledge education.

This baseline study highlights the great lack of knowledge about cervical cancer, which is consistent with other studies conducted in Ethiopia and Kenya [10, 11]. The low level of cervical cancer awareness is likely the greatest contributing factor to cervical cancer-related morbidity and mortality in Sub-Saharan African countries. The average TKS of our survey respondents was 7.84 ± 5.32 on a 23-point scale, with less than 2% scoring 20 points or more, which is also in accordance with another recently published study among women in the Lake Zone of Tanzania reporting that the median score of cervical cancer knowledge was only 16.67% and that only 17.3% scored 50% or more [12]. Our study also revealed that 40.46 and 39.04% of women were completely ignorant about warning signs and risk factors, and the highest rate of warning sign and risk factor cognitive accuracy was only 37.76%. Meanwhile, oral contraceptives, condom usage and swimming in public pools were considered risk factors for cervical cancer among quite a few participants. These misconceptions reflect women’s excessive worrying about birth control pills and devices and their mismatching with cervical cancer. Our results further support previous studies showing that women in African countries were sorely lacking in knowledge about cervical cancer, which this situation leaving much to be desired [13].

Educational level was found to be positively associated with knowledge scores, which means that this lack of knowledge could be due to the low educational level and the low coverage of cancer awareness initiatives in the country. However, in the current study, women who had obtained tertiary education were less willing to pay for screening, which contradicts previous results. We found that there was a tendency among women with higher education to be more willing to participate in free screening, even though the difference was not significant, but many of them refused to pay for screening, perhaps because they had other forms of access to free screening. As is known in Zanzibar, there are often some small-scale screening projects that are launched by different medical aid organizations at irregular intervals and that would benefit a few people, especially those who are well educated and informed. In addition, we demonstrated that women with family cancer history scored more than those without, which is consistent with previous studies [12, 14, 15]. This consistency emphasizes the influence of formal education and personal experience in understanding cervical cancer. Other factors were also found to be associated with the knowledge scores: family income, marital status, multiple deliveries and previous screening. These findings may indicate a complex relationship between health and sociodemographic factors in determining the population’s awareness of cervical cancer. Therefore, multiple policies such as public health education, social media, and interventions at healthcare facilities and by community health workers are required to improve women’s knowledge of cervical cancer, as such knowledge is a determinant of screening utilization and an important component of cervical cancer prevention.

Interestingly, our study also found that women who were reluctant to be screened scored more than those who were willing to undergo screening, which is in contrast with previous studies showing that knowledge of screening was directly and positively associated with screening intention [16, 17]. This may be because the cognitive levels, which are generally quite low, tend to exist in combination with misunderstandings of cervical cancer and screening; thus, the genuine correlation be-tween women’s awareness and their screening willingness could not be revealed in our study. However, regarding those who scored 20 points or more, almost all of them expressed a willingness to participate in a screening program even if it was self-paying. Such findings further indicate that a much-needed improvement in public awareness would be followed by a greater acceptance of screening.

As shown in our study and in previous studies, finance was an important factor affecting screening uptake. Free screening would be accepted by the majority of participants, while 34.89% (455/1304) would not pursue screening if they needed to pay for it. The main reason women re-fused to participate in non-free screening was economic. In addition, we revealed that family in-come was another noteworthy factor affecting self-paying screening. These results are unsurprising because most respondents were of low socioeconomic status and because the screening expenditure may be an added strain.

Marital status was revealed to be a significant predictor of screening uptake. Women who were married had a higher acceptance of screening than those who were divorced or unmarried, and the differences were much more significant in regard to self-paying screening. A recent study by Nwabichie CC et al. [18] also demonstrated that married women were 2 times (adjusted OR 2.257, 95% CI 1.006–4.361) more likely to have good screening uptake compared to unmarried women. This might be due to spousal support, and one study in Tanzania [19] indicated that women who received support from their husbands were more likely to receive cervical cancer screening. Another study [20] also pointed out that spouses may hinder cervical cancer screening because of their ignorance and lack of support. Therefore, more efforts are required to engage the community, including men, in promoting awareness of cervical cancer and prevention practices. Some small educational movies, health talks in communities and integration of health awareness themes into popular television and radio dramas might be effective in such promotion and prevention [21, 22].

Although schistosomiasis has been reported to be associated with cervical cancer in a few studies, an association between history of schistosomiasis infection and attitudes toward screening has not yet been reported. As shown in previous studies, schistosomiasis is an important and highly prevalent helminthic infection in which patients mainly present with vaginal discharge and abnormal bleeding in regard to female genital involvement. It is quite possible that these nonspecific symptoms make women more suspicious about cervical cancer and make them have a greater acceptance of screening. The finding in our study that women with a history of schistosomiasis were extraordinarily willing to participate in free or non-free screening also indicated that these women had suffered tremendously due to the symptoms of genital schistosomiasis and were greatly worried about malignant transformation. Another reason may be that women who have been diagnosed with schistosomiasis infection were also those who could gain access to medical resources and who had received health education and treatment; they were much more likely to participate in screening than asymptomatic women who lived lives of bare subsistence and who had no access to medical services.

Fear of screening and inconvenience were the primary concerns among the Zanzibari interviewees in all age groups. Although only 49 women thought it was not necessary for them to undergo screening, nearly all of them refused to participate in free or non-free screening, which was the main barrier to screening uptake. This result is unsurprising because they have no symptoms or discomfort, given the poor awareness of cervical cancer screening. One study conducted in Addis Ababa, Ethiopia, found similar findings, in that the most frequently mentioned barrier was women feeling healthy and thinking that such screening was unnecessary, followed by perceiving fear of positive results and the pain of the screening [10]. These findings suggest that campaigns to improve women’s cognitions of cervical cancer and screening are likely to be effective at breaking through such barriers. Our results also indicated that younger women and older women should be the focus of education. Women aged 20–49 are more likely to gather and communicate with each other because they often take their children out to play together, while younger and older women are more likely to be at home. However, according to Muslim tradition, women in Zanzibar occupy separate spaces from those of men; thus, the public places where women often gather could offer educational opportunities to raise their health awareness.

The main strength of our study is, first, the situation-based use of a mixed refinement of previous questionnaires. Data were also collected via face-to-face interviews and were double checked, minimizing the likelihood that the questions were misunderstood and that errors would be produced. Second, the study was conducted in all districts of Zanzibar, including remote rural areas, which could to some extent represent the cognitions of and attitudes toward cervical cancer and screening in the general population in Zanzibar. Third, our study is the first to indicate that schistosomiasis infection was a significant positive predictor of cervical cancer screening uptake. Moreover, our study is the first to analyze worries about cervical cancer screening among women of different age groups, suggesting that women in the younger age and older age groups receive strengthened education about screening in places where they are likely to gather according to their traditions. A limitation of this study is that most information was self-reported, which might have caused over or underestimation of certain variables. In addition, our study included all districts in Zanzibar, as some districts are more rural than others; however, due to the limited samples, the district effect on women’s willingness to participate in free or non-free screening was not checked. Lastly, our analyses were cross-sectional and implied only correlation; further research is needed to untangle the causal associations to identify key modifiable factors and to assess the effectiveness of different strategies to improve women’s awareness of and willingness to participate in cervical cancer screening in Zanzibar.

## Conclusion

Our results point to an urgent need for education and intervention to raise women’s awareness of cervical cancer and their willingness to uptake screening. Leveraging enabling factors, for example, increasing women’s awareness of cervical cancer and screening, through age-based strengthening education and publicity may help promote cervical cancer awareness and participation in the up-coming cervical cancer screening program in Zanzibar and potentially decrease the enormous local social and economic burden caused by cervical cancer.

## Supplementary information

**Additional file 1:** Cervical Cancer Awareness Survey.

## Data Availability

The datasets analyzed during the current study are not publicly available to protect the participants’ anonymity. But can be freely available from the corresponding author on reasonable request.

## Abbreviations

ANOVA: One-way analysis of variance
CAM: Cervical Cancer Awareness Measure
CCKP-64: Cervical-Cancer-Knowledge-Prevention-64
CCS: Cervical cancer Screening
CI: Confidence interval
Coef: Coefficient
OR: Odds ratio
TKS: Total knowledge score
VIA: Visual inspection with acetic acid
