# Supplementary material for: Women’s knowledge of and attitudes toward cervical cancer and cervical cancer screening in Zanzibar, Tanzania: a cross-sectional study
Source: BMC Cancer. 2020 Jan 28;20:63. doi: 10.1186/s12885-020-6528-x (PMC6988189; doi:10.1186/s12885-020-6528-x)
Supplement: Supplementary file 1 — Additional file 1: Cervical Cancer Awareness Survey. [file 12885_2020_6528_MOESM1_ESM.docx]

**Appendix A**

| **Background information** | | | | | | | | | | |
| --- | --- | --- | --- | --- | --- | --- | --- | --- | --- | --- |
| **Address** |  | | | **Telephone** | |  | | | **Age** | |
| **G**  **P**  **A** | **Marriage**  Married/cohabiting Divorced/widowed  single | | | **Religion**  Muslim  Jesus  other | | **Education**  No formal  Primary  Secondary  Tertiary | | | **Husband's education**  No formal  Primary  Secondary  Tertiary | |
| **Family income**  High  Middle  Low | **Drinking water**  River water  Well water  Tap water  Pure water  Other | | | **Spice tea**  Often  Occasionally  Rare  Never | | **Coffee**  Often  Occasionally  Rare  Never | | | **Soda**  Often  Occasionally  Rare  Never | |
| **Eating Habit** Drink/Smoke/Both no  Vegetables Often/Occasionally/R/N  Fruits Often/Occasionally/R/N  Meat Often/Occasionally/R/N  Sea food Often/Occasionally/R/N  Milk Often/Occasionally/R/N  Beans Often/Occasionally/R/N  Bread Often/Occasionally/R/N  Rice Often/Occasionally/R/N  Manihot Often/Occasionally/R/N  Oily Fried or grilled Often/Occasionally/R/N | | | | **Menarche(y)**  <12  12-15  16-19  ≥20  Other  **Menstruation**  Normal  Hypomenorrhea  Dysmenorrhea  Excessive Irregular  Menostasis | | **Abnormal Pregnancy**  No  Abortion  Stillbirth  Birth defect  Child death  Mole  Complication  Other | | | **Residency(y)**  <1  1-5  6-15  >15 | **Sleep**  Very good  Fairly good  Not good or bad  Fairly bad  Very bad  **Pressure**  None  Mild  Moderate  Severe  Extreme |
| **Age of husband**  **Marriage age**  **Consanguineous marriage**  Yes No Don't know  **Partner's urogenital disease**  Yes No Don't know  **First sex age（y）** | | | | **Birth control**  No  Condom  COC  IUD  Tube ligation  Other | | **Freq. of sexual life**  > Twice/W  1-2 times/W 1-3 times/M  <Once/M | | **Previous Examination** Gynecologic examination  Pap smear  Pelvic ultrasound  Cervix /endometrial biopsy  Other  Don't know | | |
| **History of disease**  None  Yes  Don't know | | **History of surgery**  None  Yes  Don't know | | | **Medication**  None  Yes  Don't know | | **Genetic disease**  None  Yes  Don't know | | | **Family cancer**  None  Yes  Don't know |
| **Willing for free screening**  Yes No Don't know  **If it is not free, will you do?**  Yes No Don't know | | | **Do you have these worries?**  Fear to give a Pap-smear/ Long appointment queues  /Unsuitable reception hours/Clinic is far away  /A recent health control at a gynecologist / Removed uterus  /It is not necessary for me/Other reasons/None | | | | | | | |

| **Awareness** | |
| --- | --- |
| **Have you heard of cervical cancer?**  Yes No Don't know | **Can cervical cancer be a terminal illness?**  Yes No Don't know |
| **Is there any effective method that significantly reduces the risk?**  Yes No Don't know | **Do you think this disease could affect you in the future?**  Yes No Don't know |
| **The following may or may not be warning signs for cervical cancer. Tick the one(s).**  Vaginal bleeding between periods  Persistent lower back pain  Persistent vaginal discharge  Discomfort or pain during sex  Heavier or longer menstrual periods  Persistent diarrhea  Vaginal bleeding after the menopause  Persistent pelvic pain  Vaginal bleeding during or after sex  Blood in the stool or urine  Unexplained weight loss  None of them Don't know | **If you had a symptom that you thought might be a sign of cervical cancer how soon would you go to see your doctor?**  Instantly  In three days  In a week  In a month  In 6 month  In one year  More than one year  Never Don't know  **Women at which age is most likely to suffer from cervical cancer?**  20-29 30-49 50-69 70 or above unrelated to age None of them Don't know |
| **The following may or may not risk factors of cervical cancer. Tick the one(s).**  Infection with HPV Smoking  Hypo immune function  Long term of contraceptive pill use  Sexually Transmitted infection  Sex at a young age  Many sexual partners  Many children  Sexual partner with many other partners  Not going for regular smear(Pap) tests  Rare health control  Genetic factors  Miscarriages and abortions  Use of condoms  Public swimming pools  None of them Don't know | **How confident are you that you would notice a cervical cancer symptom?**  Not at all Not very Fairly Very  **Have you heard of cervical cancer screening?**  Yes No Don't know  **Have you done screening before?**  Yes No Don't know  **Did you do with /without symptoms?**  Yes  No Don't know  **Do you know the main purpose of cervical screening?**  Yes No Don't know  **At which age do you think is appropriate for the female to start cervical screening?**  <18 18-20 21-29 30-39 40-49 50-59 60 or above Don't know |
| **Do you know other messages about screening? Interval/ pain/method/ effect/fees?** | **Now that the interview is over, would you like to ask any questions? Or any comments?** |
